# The Yin and Yang of epigenetics in the field of nanoparticles

**DOI:** 10.1039/d1na00682g

**Published:** 2022-01-10

**Authors:** Elettra Musolino, Christina Pagiatakis, Simone Serio, Marina Borgese, Federica Gamberoni, Rosalba Gornati, Giovanni Bernardini, Roberto Papait

**Affiliations:** Department of and Life Sciences, Insubria University Via Dunant 3 21100 Varese Italy roberto.papait@uninsubria.it; Department of Cardiovascular Medicine, Humanitas Research Hospital Rozzano MI Italy; Department of Biomedical Sciences, Humanitas University Via Rita Levi Montalcini 4 20090 Pieve Emanuele MI Italy

## Abstract

Nanoparticles (NPs) have become a very exciting research avenue, with multitudinous applications in various fields, including the biomedical one, whereby they have been gaining considerable interest as drug carriers able to increase bioavailability, therapeutic efficiency and specificity of drugs. Epigenetics, a complex network of molecular mechanisms involved in gene expression regulation, play a key role in mediating the effect of environmental factors on organisms and in the etiology of several diseases (*e.g.*, cancers, neurological disorders and cardiovascular diseases). For many of these diseases, epigenetic therapies have been proposed, whose application is however limited by the toxicity of epigenetic drugs. In this review, we will analyze two aspects of epigenetics in the field of NPs: the first is the role that epigenetics play in mediating nanotoxicity, and the second is the possibility of using NPs for delivery of “epi-drugs” to overcome their limitations. We aim to stimulate discussion among specialists, specifically on the potential contribution of epigenetics to the field of NPs, and to inspire newcomers to this exciting technology.

## Abstract 2

Evaluating the role of epigenetics in the field of NPs: first, the role of epigenetics in mediating nanotoxicity, and second, the possibility of using NPs for delivery of “epi-drugs” to overcome their limitations.

## Graphical abstract

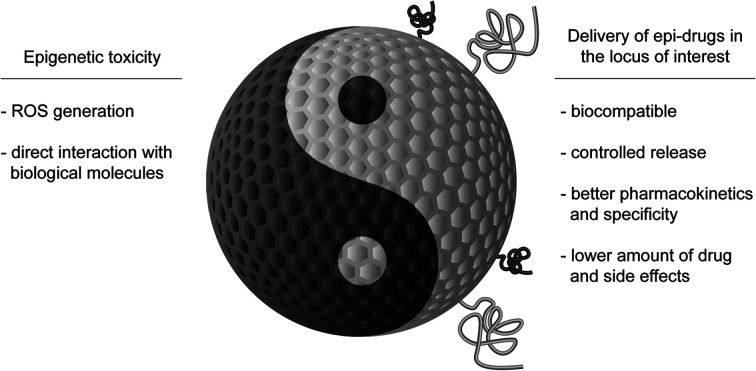

## Introduction

There is a general consensus in defining nanoparticles (NPs) as small particles ranging from 1 to 100 nm in size, though, quite often, particles with a diameter of a few hundreds of nm are still considered NPs. More precise and comprehensive definitions of NPs and nanomaterials have been given by different organizations, mainly for regulatory purposes, although, in this regard, a single internationally accepted definition has not yet been reached.^1^ NPs can be grouped as naturally occurring NPs (natural NPs, NNPs) and NPs that are produced (intentionally or not) by human activities (anthropogenic NPs, ANPs). Among NNPs, we can consider those produced by volcanic eruptions, forest fires and dust storms, however, viruses and exosomes can also be considered NNPs.^2^ Among ANPs, those present in welding fumes, diesel exhaust, cigarette smoke and building demolition, as well as nanoplastics, are examples of incidental NPs (INPs). Instead, NPs that are intentionally produced and designed with specifically tailored chemical and physical properties are called engineered NPs (ENPs), and are increasingly pertinent in numerous applications in various fields including industrial, electronic, construction, cosmetic, and environmental fields.^3^ Finally, the increasingly widespread use of NPs in the biomedical field for diagnostics (*e.g.*, imaging and medical biosensors), regenerative medicine (*e.g.*, tissue engineering) and therapeutics (*e.g.*, drug delivery) is of critical importance.

The development of a wide range of NPs for numerous applications has increased our exposure to them *via* different routes (*i.e.*, inhalation, ingestion and skin absorption), which, in turn, can cause short- and long-term toxicity. Considering the importance that NPs are acquiring in the biomedical field, another important route of uptake that should be considered is intravenous injection as a result of the presence of NPs in contrast agent imaging and in drugs. Thus, the assessment of the toxicological risk associated with NPs must be considered as an integral part of their design and production in order to guarantee safety to the environment, consumers and workers.^4,5^ In this context, a specific branch of the toxicological sciences, nanotoxicology, took shape. In the last decade, considerable progress has been made in the field of nanotoxicology. In particular, the advent of new technologies has allowed for the combination of canonical methods (*e.g.*, cell-based *in vitro* assays) with advanced multidisciplinary methods (*e.g.*, computational models, green algorithms and electrochemical approaches) to improve the reliability of both preclinical and clinical assessments regarding the toxicity of nanoparticles and others nanomaterials.^6^ Despite this, new approaches are needed for a better mechanistic understanding of how nanomaterials can perturb biological systems and, eventually, lead to adverse effects.^7^ In this regard, a systems biology-oriented approach, aimed at a holistic understanding of the mechanisms of interaction between nanomaterials and living systems, might be a winning bid.^7^ To this aim, global “omics” approaches such as genomics, transcriptomics, proteomics, metabolomics and, more recently, epigenomics have been utilized. Epigenetics are a set of mechanisms (*e.g.*, DNA methylation, histone modifications, ATP-dependent chromatin-remodeling complexes and non-coding RNAs) that define the status of gene expression without changes in the DNA sequence, and whose aberrant alterations can be the cause of serious diseases (*e.g.*, cancer, neuronal disorders and cardiovascular diseases).^8–10^ Since epigenetic mechanisms have been shown to be perturbed in response to various environmental factors, thus influencing cellular activity, it is necessary to evaluate epigenetic regulation as a potential pathway through which NPs interfere with cellular function.^11^

Another important application of epigenetics in the field of NPs regards the generation of less toxic and more efficient epigenetic drugs.^12^ Since epigenetic mechanisms are important in the etiology of various diseases, using epigenetic therapies may be resolutive in their cure. However, a very pertinent limitation of current epigenetic drugs is their toxicity, due to the ubiquitous expression of their targets. Thus, a precise targeting of epi-drugs would be highly desirable. Indeed, the specific delivery of an agent to a specific disease site is a major challenge in pharmacology: in fact, only a small percentage of the dose arrives to the organ of interest and, even less, targets the desired cell type. The usual solution is to increase the dose to assure a sufficient amount at the target site. This causes side effects and general toxicity, which often severely limits the clinical use of otherwise promising molecules. One approach to directing drugs to the locus of interest is to use NPs as delivery systems. In recent years, several NPs have been developed that are able to actively or passively transport drugs to the locus of interest by binding them to their external surface or encapsulating them. Active targeting takes advantage of the recognition of specific ligands (*e.g.* peptides, carbohydrates, antibodies and vitamins) present on nanoparticles by cellular receptors expressed mainly or exclusively on the site of interest, whereas passive targeting takes advantage of the physical–chemical characteristics of the nanoparticles (*e.g.*, size, shape and charge) and of the characteristics of the target.^13^ For example, NPs that possess cell-specific surface ligands, such as liposomes, lipid nanoparticles (LNPs) and polymeric nanoparticles, have proved to have modest to accurate active targeting performance,^14–16^ whereas intravenously injected magnetic NPs have been shown to have remarkable passive targeting performance, as they can be captured and confined in a desired area (*e.g.*, a solid tumor) by a static magnetic field. Many studies have also been carried out in order to investigate how the drug could be released from the nanoparticles once they have reached the site of interest. These studies have led to the creation of different types of nanoparticles capable of releasing drugs by exploiting variations in pH or temperature, or by using magnetic fields, electric fields or ultrasound.^17–21^ In principle, these approaches can increase the delivery efficiency, reduce the total amount of drug administered, and therefore limit off-target and undesired effects.^22^ For example, assays carried out in HeLa cells have displayed a temperature-dependent controlled release of the anticancer drug doxorubicin incapsulated in temperature responsive fluorescent nanoparticles (TRFNPs),^23^ whereas biocompatible nanoparticles of magnetic iron oxide (IONPs) loaded with doxorubicin, following the application of an external magnetic field, have shown an enhanced penetration and an increased therapeutic response in glioblastoma multiforme cell lines.^24^ The possibility to use NPs to deliver drugs is supported by many studies published over the last few years that have revealed the possibility to use NPs as vectors for several anti-cancer drugs (*e.g.*, gold NPs conjugated with doxorubicin for the treatment of ovarian cancer),^25,26^ and also for viral and non-viral gene (*e.g.*, magnetic silica NPs conjugated with TK/GCV for the treatment of hepatocellular carcinoma)^27–29^ and antibiotic (*e.g.*, silver NPs conjugated with ampicillin against *K. pneumonia* and *E. coli*)^30,31^ delivery. To date, in fact, the US Food and Drug Administration (FDA) has approved about twenty nano-drugs capable of treating several diseases.^32^

**Fig. 1:**
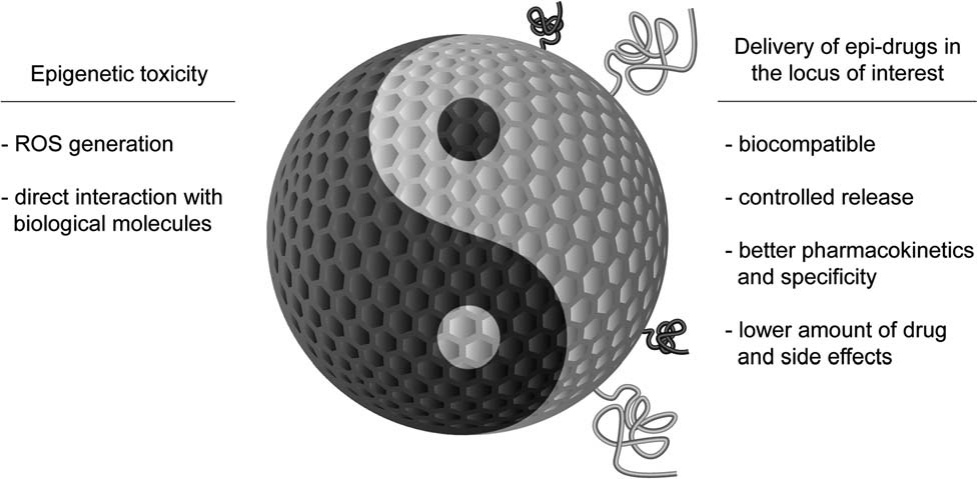
The aspects of epigenetics in the field of nanoparticles.

Here, we will discuss two aspects that combine epigenetics and nanotechnology. On one side, we will consider the role of epigenetic mechanisms in mediating nanotoxicity, and, on the other side of the coin, the possibility to use NPs for delivering epi-drugs to overcome the limitations that are currently hindering their use (Fig. 1). We aim to stimulate discussion amongst specialists on the progress that the study of epigenetics in the field of nanotechnology could bring to both fields and to inspire newcomers to this exciting technology.

## Epigenetic mechanisms

**Fig. 2:**
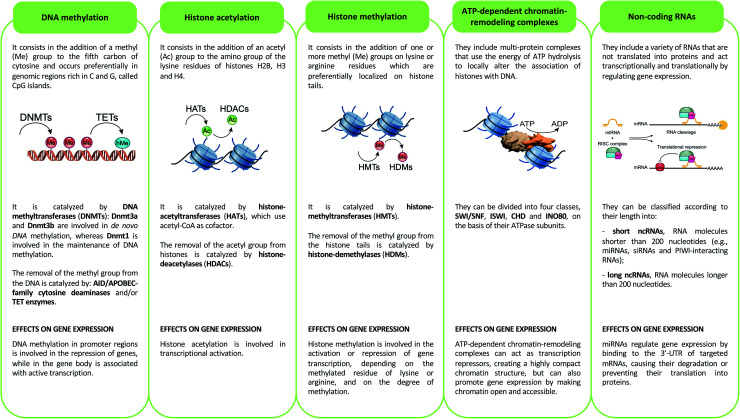
Description of the main epigenetic mechanisms and their effects on gene expression.

Epigenetics mechanisms include DNA methylation, covalent histone modifications, ATP-dependent chromatin-remodeling complexes and non-coding RNAs. These mechanisms cooperate in defining the transcription status of each one of more than 200 cell types that make up the human body, acting by either modulating the accessibility of genes and recruitment of transcription machinery, or by regulating the half-life of mRNA^33^ (Fig. 2).

### DNA methylation

DNA methylation consists of methylation of the fifth carbon of cytosine (5-methylcytosine, 5mC). This epigenetic mechanism is involved in the repression of genes when its deposition occurs in the promoter regions of these genes, while it is associated with active transcription when it is present in the gene body.^34^ 5mC occurs preferentially in genomic regions rich in C and G, called CpG islands, and is catalyzed by DNA methyltransferases (DNMTs).^35^ DNA methylation promotes transcriptional repression by binding methyl-binding proteins (MBDs) and zinc-finger proteins. These proteins promote the formation of a closed chromatin structure, not accessible to transcription factors and transcription machinery, through binding with repressor complexes.^36^

DNA methylation is a reversible epigenetic mechanism, inasmuch, the methyl group of 5mC can be removed through an active process that requires the deamination (mediated by AID/APOBEC-family cytosine deaminases) and/or oxidation (performed by ten–eleven translocation enzymes) of 5-methylcytosine that leads to the formation of 5-hydroxymethylcytosine (5hmC) that, in turn, is converted in 5-formylcytosine (5fC) and 5-carboxylcytosine (5caC): both of these modified bases are replaced with cytosines by the base excision repair (BER) pathway.^37^ Of note, DNA hydroxymethylation can also act as an epigenetic mark able to promote transcription when this modification occurs in the gene body and enhancer regions, genetic elements that have key roles in modulating transcription during development,^38^ and in several diseases such as congenital malformations, heart disease, cancer and intellectual disabilities.^39^

### Histone modifications

Histone modifications are covalent modifications (*e.g.*, acetylation, methylation, phosphorylation, ubiquitylation and sumoylation) able to regulate gene expression by defining chromatin conformation or by recruiting proteins involved in transcription. Histone modifications occur on specific amino acid residues, which are most often found on the amino-terminal tails of histones. To date, the most studied histone modifications are acetylation and methylation.^40^

**Fig. 3:**
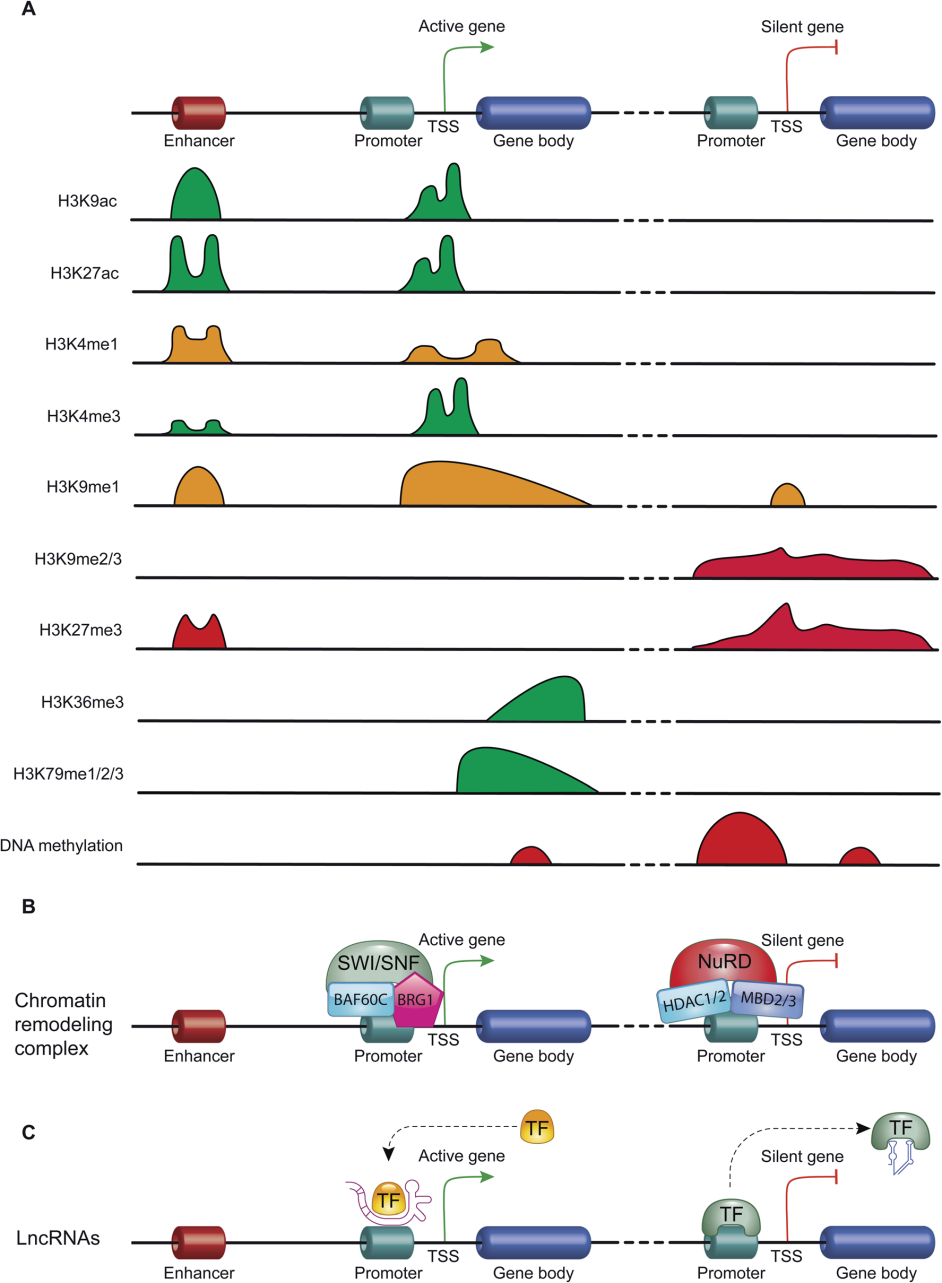
The mechanisms of action of the main epigenetic mechanisms: (A) the genomic distribution of the main histone modifications across transcriptional regulatory elements (*e.g.*, promoter, enhancer and gene body) of active and repressed genes. Schematic diagram showing the enrichment of the histone H3 modifications that promote transcription activation (green) and those that promote transcription repression (red). The enrichment of H3K4me1 and H3K9me1, a histone mark involved in both transcriptional activation and repression, is indicated in orange. (B) Two examples of chromatin remodeling complex with opposite effects on transcription, the chromatin remodeling complex SWI/SNF promotes the gene transcription, while NuRD complex leads the transcription repression. (C) The mechanism of action of lncRNAs on gene transcription: schematic diagram showing that this class of ncRNAs can act as transcription activators promoting the recruitment of transcription factors (TFs) on promoter regions, or as transcription repressors by displacing TFs away from promoters.

Histone acetylation is regulated by the opposite action of two families of enzymes: histone-acetyltransferases (HATs) and histone-deacetylases (HDACs). HAT enzymes, using acetyl-CoA as cofactors, bind an acetyl group to the amino group of the lysine residues of histones H2B, H3 and H4.^41^ By neutralizing the positive charge of the amino acid, these enzymes weaken the electrostatic interaction between histones and DNA. In this way, chromatin becomes more accessible to transcription factors and RNA polymerase and, therefore, transcription is promoted.^42^ Moreover, acetylated histones are docking sites for the bromodomains and extra-terminal domains (BET), that act as positive regulators of transcription^43^ (Fig. 3A). In contrast, HDAC enzymes are able to deacetylate lysine residues, restoring their positive charge: they act as transcriptional repressors, stabilizing chromatin architecture.^41^

Another important histone modification is methylation. The level of histone methylation is the result of the activity of two classes of enzymes: histone-methyltransferases (HMTs) and histone-demethylases (HDMs). The HMT enzymes add one or more methyl groups on lysine or arginine residues that are preferentially localized on histone tails.^44^ Lysines can be mono-, di- or tri-methylated, while arginines can be mono- and di-methylated. The methylation of arginines can be symmetric or asymmetric. In contrast, HDM enzymes catalyze the demethylation of histone tails. Histone methylation is able of either activating or repressing gene transcription, depending on the methylated residue of lysine or arginine, and on the degree of methylation. For example, high levels of tri-methylated histone H3 at lysines 4 and 36 (H3K4me3 and H3K36me3), di-methylated histone H3 at lysine 79 (H3K79me2) and mono-methylated histone H3 at lysines 9 and 20 (H3K9me1 and H4K20me1) are associated with transcription activation. On the contrary, elevated levels of H3K9me2, H3K9me3, H3K20me3 and H3K27me3 are associated with transcriptional repression^44,45^ (Fig. 3A).

Another important histone modification is phosphorylation. Histone phosphorylation occurs mainly, but not limited, on the residues of tyrosine, threonine and serine located on the N-terminal tails. This process is mediated by two different classes of enzymes: kinases and phosphatases. The former functions by transferring a phosphate group from ATP to the hydroxyl group of the amino acid, introducing a negative charge at the nucleosome level and, therefore, promotes relaxation of chromatin, while the latter mediates dephosphorylation of these sites.^40^ Histone phosphorylation, in addition to having a role in DNA damage repair, also has a role in transcription regulation and in chromatin compaction. Like other histone modifications, the same phosphorylated residues can have different consequences on chromatin structure, depending on the context. For example, phosphorylation of H3S10 and H3S28 can be involved both in chromatin condensation associated with mitosis and meiosis, and in chromatin relaxation associated with the activation of transcription.^46^

Histone ubiquitylation occurs by means of the sequential action of three enzymes (E1-activating, E2-conjugating and E3-ligating enzymes), which add ubiquitin, a small protein of 76 amino acids, to lysine residues. Lysines can be monoubiquitylated or polyubiquitylated, and the histones most affected by this modification appear to be H2A and H2B: the impact of this histone modification depends on which histone becomes ubiquitinated. The mono-ubiquitination of H2A at lysine 199 (H2AK119ub1) promotes transcription repression, while the monoubiquitylation of H2B at lysine 123 (H2BK123ub1) regulates transcription initiation and elongation. Like other histone modifications, ubiquitylation is a dynamic process, and can also be removed. This occurs through the action of the de-ubiquitin isopeptidase.^40^

The enzymes E1, E2 and E3 involved in ubiquitylation are also able to bind a small ubiquitin-like molecule on the lysine residues of histones. This process is called sumoylation and acts on all four histones (H2A, H2B, H3 and H4) by antagonizing the processes of acetylation and ubiquitylation. Therefore, sumoylation is a histone modification mainly associated with gene silencing.^40^ However, recent findings suggest that histone sumoylation can also act as a signal for recruitment of factors involved in transcriptional activation.^47^

### ATP-dependent chromatin-remodeling complexes

ATP-dependent chromatin-remodeling complexes are multi-protein complexes that use the energy of ATP hydrolysis to locally alter the association of histones with DNA.^48^ In particular, this mechanism of gene expression regulation aims to control DNA-binding site accessibility to various transcription factors. ATP-dependent chromatin-remodeling complexes can be divided into four classes, SWI/SNF, ISWI, CHD and INO80, on the basis of their ATPase subunits. These four remodeler families are involved in both transcriptional activation and repression.^49–51^ ATP-dependent chromatin-remodeling complexes can act as transcription repressors, creating a highly compact chromatin structure that is unable to accommodate transcription factors, but can also promote gene expression by making chromatin open and accessible. Members of the SWI/SNF family, for instance, promote transcriptional activation through a mechanism that involves the sliding of nucleosomes, the removal of H2A and H2B dimers, or the removal of all histone octamers from DNA^52^ (Fig. 3B).

### Non-coding RNAs (ncRNAs)

Non-coding RNAs (ncRNAs), which include a variety of RNAs that are not translated into proteins, act transcriptionally and translationally by regulating gene expression. Non-coding RNAs are classified according to their length: short ncRNAs (RNA molecules shorter than 200 nucleotides) and long ncRNAs (RNA molecules longer than 200 nucleotides).^53^ Short ncRNAs include microRNAs (miRNAs), small interfering RNAs (siRNAs) and PIWI-interacting RNAs. Among the non-coding RNAs, those currently most studied are miRNAs, small RNAs with a length of 19–25 nucleotides. MicroRNAs are able to regulate gene expression by binding to the 3′ untranslated region (3′-UTR) of targeted mRNAs, causing their degradation or preventing their translation into proteins. In this way, miRNAs regulate proliferation, differentiation, survival and cell death. Instead, lncRNAs are a more heterogeneous group of ncRNAs which regulate gene expression using various mechanisms, and have been shown to be key in regulating development, cellular homeostasis and also pathogenesis^54,55^ (Fig. 3C).

**Fig. 4:**
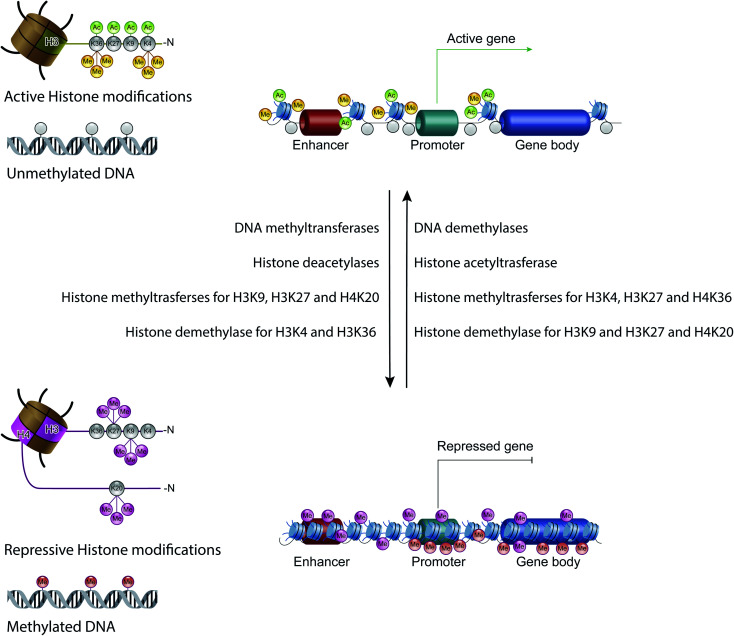
The epigenetic code: schematic diagram showing the combination of the main epigenetic marks that define the transcriptional status of a gene: transcribed genes have an epigenetic signature characterized by unmethylated DNA and high levels of acetylated histone H3 on lysine (K) 4, 9, 27 and 36, and of trimethylated histone H3 on lysine (K) 4 and 36. Instead, repressed genes possess high levels of trimethylated histone H3 on lysine (K) 9 and 27, and of trimethylated histone H4 on lysine 20.

Therefore, except for the ncRNAs that act at the post-transcriptional level on mRNAs, DNA methylation, histone modifications and ATP-dependent chromatin-remodeling complexes control gene expression by modulating the architecture of chromatin and by controlling DNA-based biological processes, such as the accessibility of transcription factors to promoters and also transcription elongation. Importantly, all these epigenetic marks act in a coordinated fashion when defining the transcriptional status of a gene^56,57^ (Fig. 4).

## Epigenetic toxicity: the Yin of epigenetics in the field of NPs

Epigenetic mechanisms mediate the effects of environmental cues on phenotype through the regulation of transcription: alterations of these mechanisms have been shown to be deregulated during important cellular processes such as cell cycle, DNA repair, and cell differentiation, leading to several human diseases (*e.g.*, cancer, neuronal disorders, and heart failure) and developmental disorders.^58^ Despite this, little is known about the role of epigenetic drugs and NPs in mediating cellular toxicity. Interestingly, most of the studies regarding epigenetic toxicity of NPs limit themselves in defining the effects that they have on genomic distribution of certain epigenetic marks, such as DNA methylation and histone acetylation, or in promoting alterations of expression of various miRNAs. Importantly, it has not yet been defined whether these epigenetic changes result in cellular toxicity, and if NPs themselves are directly involved in promoting epigenetic changes.

**Fig. 5:**
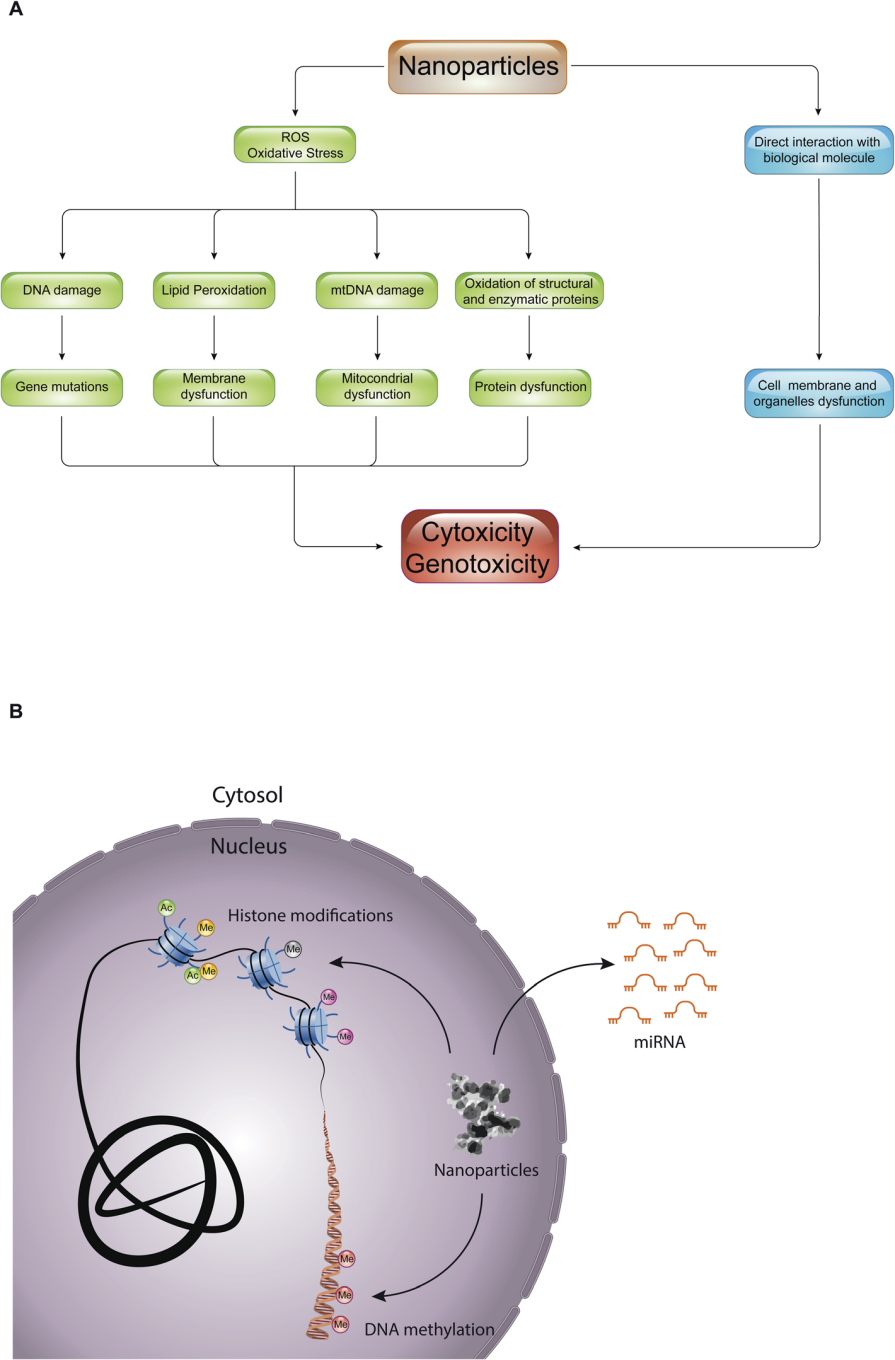
Nanotoxicity at the epigenetic level: (A) diagram of pathways that promote toxicity of NPs in the cell. (B) Schematic representation of NPs that can cause epigenetic toxicity by interfering with the main epigenetic mechanisms, such as DNA methylation, histone modifications and micro RNAs (miRNAs).

The toxic effect of NPs, with which we constantly come into contact through the use of textile products, food packaging, dietary supplements, electronic devices, hygiene products and cosmetics, can result in production of reactive oxygen species (ROS) or in a direct interaction with biological molecules such as lipids, proteins, and nucleic acids (Fig. 5A). In the first case, the overproduction of ROS causes lipid peroxidation, mitochondrial dysfunction, DNA damage and protein denaturation, leading to cytotoxicity and genotoxicity. For example, 14 nm silica NPs induce apoptosis in a dose-dependent manner in HepG2 human liver cancer cell line, promoting the production ROS.^59^ The exposure of A549 human lung epithelial cells to silver NPs (AgNPs) for 24 hours caused cell cycle arrest in the G_2_/M phase, and gave rise to an excessive production of reactive oxygen species able to modify the expression of over 1000 genes.^60^ In addition, numerous studies have shown that ROS are responsible for the toxicity of gold-cobalt, copper oxide, zinc oxide, and titanium dioxide NPs.^61–65^ The quantity of ROS produced, and thus the cell damage caused, depends on the physical–chemical properties of NPs (such as size, shape, charge, chemical composition, solubility, and ability to form aggregates or agglomerates), on environmental factors that can influence their activity, and on cellular type with which NPs come into contact. With equal chemical composition, for example, the smaller the NPs, the greater their ability to react with biological molecules producing reactive oxygen species.^4^ Instead, in the second case, the interaction of NPs with biological molecules altered the function of the cell membrane and of organelles (*e.g.*, lysosome, mitochondria or cell nucleus). Quantum dots (semiconductor NPs) release cadmium ions (Cd^2+^) that localize in the lysosomes, causing lysosomal enlargement and intracellular redistribution.^66^ Once in the cytoplasm, NPs can penetrate the nucleus by diffusion through the nuclear pores, or accidentally, during the mitosis process.^67^ In the nucleus, they can bind directly to DNA and nuclear proteins, perturbing their function. For instance, SiO_2_ NPs enter into the nucleus, where they trigger the formation of nuclear aggregates containing proteins important for nuclear function, such as histones, CREB-binding protein (CBP) (transcription activator), and topoisomerase I (an enzyme involved in the over- or under-winding of DNA during DNA replication, transcription, chromatin assembly, DNA repair and recombination). It was proposed that, through the formation of these aggregates, SiO_2_ NPs have a negative impact on cell proliferation thus inhibiting DNA replication and transcription.

The first studies conducted on the epigenetic effects of NPs regard DNA methylation. These studies have shown that NPs can induce changes in DNA methylation patterns: the treatment of the HaCaT cell line (human epidermal keratinocytes) with silicon dioxide NPs (SiO_2_ NPs) causes, on the one hand, a hypomethylation associated with a decrease in the levels of expression of Dnmt1, Dnmt3a and MBD2, and on the other hand, an increase of DNA methylation in the promoter of poly(ADP-ribose) polymerase (PARP-1) gene, causing it repression. PARP-1 plays a key role in the early cellular response to DNA damage, and its inactivation leads to genomic instability and apoptosis.^68^ Repression of PARP-1 through DNA methylation was also described in adenocarcinoma cells treated with TiO_2_.^69^

Decreases in the global DNA methylation profile and DNA methyltransferase activity (Dnmt1, Dnmt3a and Dnmt3b) were also found in MRC5 lung fibroblasts treated with different concentrations of titanium dioxide (TiO_2_) and zinc oxide (ZnO) NPs.^70^ On the contrary, carbon-based nanoparticles (fullerene, multi-walled carbon nanotubes and single-walled carbon nanotubes) treatment causes a global increase in DNA methylation in A549 human lung cells.^71^ Moreover, it was suggested that multi-walled carbon nanotubes cause pulmonary toxicity promoting an inflammatory process by hypomethylation of the promoter of TNFα, a key gene of the immune response, and also promoting fibrotic onset through promoter methylation of THY-1, a gene involved in idiopathic pulmonary fibrosis.^72^ Finally, the intra-tracheal administration of 60 nm gold NPs (AuNPs) in BALB/c mice caused hypermethylation of the promoters of genes coding for ataxia telangiectasia mutated (ATM), cycle-dependent kinase (Cdk) and glutathione reductase (Gsr), and hypomethylation in the promoter of glutathione peroxidase (Gpx)^73^ in lung tissue. AuNPs also promote a hypermethylation of the promoter region of the tumor suppressor protein P53 (trp53) that depends on the dose and size of the nanoparticle.

The effects of NPs on histone modifications have also been previously described. Short term exposure to cadmium telluride quantum dots (QDs) causes global histone hypoacetylation of MCF-7 and, consequently, chromatin condensation in the MCF7 human breast cancer cell. This histone modification change was reverted by treatment with trichostatin A, a histone-deacetylase (HDAC) inhibitor. However, it was not clear whether the histone hypoacetylation was the consequence of a direct effect of this NP on specific mechanisms involving histone acetylation.^74^ Interestingly, silver NPs (AgNPs) cause a significant reduction in the levels of global methylation of histone H3 in mouse erythroid cells by binding to histone H3 and H4, which protects them from methylation catalyzed by histone methyltrasferases.^75^ Moreover, epithelial cells of the small human airway treated with gold NPs (AuNPs), showed a decrease in the trimethylation of lysine 27 on histone H3 (H3K27me3).^67^ Regarding histone phosphorylation, silver NPs (AgNPs) are able to induce phosphorylation of histone H3 on serine 10 (H3S10) in A549 human lung adenocarcinoma cells, resulting in activation of the entire MAPK cascade by Ag ions released by NPs.^76^

While the above-mentioned studies investigated the impact of NPs on a single epigenetic modification, there are other studies that have aimed at studying the effect of NPs on the crosstalk of histone modifications. It was found that arsenic trioxide NPs (As_2_O_3_ NPs) cause a decrease in the methylation of histone H3 on lysine 9 (H3K9) and an increase in both the phosphorylation of histone H3 on serine 10 (H3S10) and the acetylation of histone H3K14 in two human prostate cancer cell lines (LNCaP and PC-3).^77^ Moreover, the treatment of HaCaT human epidermal keratinocytes with zinc NPs (ZnO NPs), in addition to causing the arrest of the cell cycle at the G_2_/M checkpoint, led to an increase of di-methylated lysine 9 (H3K9me2) of histone H3, and a simultaneous decrease in histone H4 acetylation of lysine 5 (H4K5), defining an epigenetic signature associated with chromatin condensation. These epigenetic changes were accompanied by an increase of expression of G9a and GLP, two histone methyltransferases that catalyze the mono- and di-methylation of lysine 9 of histone H3, and a down regulation of expression of several histone acetyltransferases such as GCN5, P300 and CBP. These epigenetic changes could be the result of the formation of ZnO NP aggregates in perinuclear regions, which might directly interact with the nucleus by perturbing its structure, or of production of ROS induced by these aggregates.^78^

Numerous studies have therefore analyzed the effect of nanoparticles on DNA methylation or histone modifications. However, since DNA methylation is an epigenetic modification that acts in a coordinated manner with histone modifications in defining the transcriptional status of genes, it would be interesting to study these two epigenetic modifications simultaneously when analyzing the effect of nanoparticles. Furthermore, to date, as far as we know, studies regarding the effect of NPs on chromatin remodeling complexes have not been carried out, whereas studies on the epigenetic effects of NPs in terms of deregulation of non-coding RNAs and, in particular, of microRNAs, have been increasing. It has been found that treatment with silver NPs (AuNPs) in human T Jurkat cells altered the expression of 63 miRNAs. Among these, miR-504, miR-33 and miR-302 could have a role in mediating DNA damage and apoptosis induced by these NPs.^79^ A study conducted in PC12 neuronal cells subjected to the action of super-magnetic iron dioxide NPs (SPIONs), a material widely accepted for performing magnetic resonance, has demonstrated a significant change in the expression of some cellular miRNAs. This study suggested that miRNAs down-regulated the expression of the *N*-methyl-d-aspartate receptor (NMDAR), and key genes of neuronal plasticity, outgrowth and survival, and its repression caused neuronal apoptosis.^80^

In A549 human lung cells, treatment with TiO_2_ NPs caused a significant downregulation of miRNA-21 and miRNA-30a, important regulators of the autophagy process,^81^ while the treatment of NIH3T3 murine fibroblasts with CdTe quantum dots was able to induce significant changes in the expression of 51 miRNAs, 16 of which were downregulated and 35 upregulated.^82^ A significant number of studies have also been conducted to evaluate the *in vivo* effects of NPs on miRNAs. For example, by exposing adult female C57BL/6BomTac mice to surface coated titanium dioxide NPs (TiO_2_ NPs), significant changes were observed in the expression of 16 miRNAs in the lung. Of these, miR-1 was 6-fold upregulated, miR-449a 2.6-fold, and miR-135b 60-fold, compared to mice that were not exposed to NPs.^83^ It was also found that 100 nm gold NPs (AuNPs) were able to induce alteration of expression of several miRNAs (*e.g.*, upregulation of Let-7a and miR-183) in mouse fetal liver and lung.^84^ There are also studies that showed that some NPs are able to induce changes to levels of some miRNAs in the blood, suggesting that, based on the content in circulation, they could be a biomarker of toxicity for this type of NP. Gold NPs in mice caused a change in the expression profile of miRNAs in blood cells in a time-dependent manner, and treatment of mice with 70 nm diameter silica NPs (nSP70) caused liver damage that was accompanied by an increase in the amount of two liver-specific miRNAs (miR-122 and miR-192). Since miR-122 levels varied more than miR-192, and because the sensitivity of this miRNA for liver damage was equal to that of other markers of liver damage, it was proposed that miR-122 be a new biomarker of liver damage induced by the action of silica NPs and other nanomaterials^85^ (Fig. 5B).

To date, several studies have therefore found a correlation between the administration of nanoparticles and alterations of certain epigenetic marks (Table 1). However, the mechanisms of action by which the nanoparticles could induce epigenetic toxicity are not yet fully understood and require further investigation. On one hand, NPs could induce these epigenetic changes indirectly through the overproduction of ROS. Numerous studies have, in fact, demonstrated that ROS are able to cause epigenetic modifications by acting both directly and indirectly on DNA, histones, and miRNAs.^86^ On the other hand, NPs could also induce epigenetic toxicity through direct interaction with biological molecules, such as DNA, histones, and miRNAs. Finally, it would be interesting to investigate in more detail how the physical–chemical characteristics of nanoparticles, such as size, shape, charge, *etc.*, can affect the type or degree of alteration of epigenetic mechanisms.

**Table 1:** List of the main studies regarding nanotoxicity at the epigenetic level

	Nanoparticles	Nanoparticles size	Dose and exposure time	Biological model	Epigenetic effect	Year	Reference
Changes in DNA methylation	Silicon dioxide (SiO_2_)	1–5–15 nm	2.5–10 μg ml^−1^ for 24 h	HaCaT human epidermal keratinocytes	Hypermethylation of the PARP-1 promoter	2012	68
	Titanium dioxide (TiO_2_)	22.1 nm	6.25–100 μg ml^−1^ for 24 h	A549 human alveolar epithelial cells	Hypermethylation of the PARP-1 promoter	2015	69
	Titanium dioxide (TiO_2_) and zinc oxide (ZnO)	<100 nm	0.125–8 μg ml^−1^ for 24–72 h	MRC5 lung fibroblasts	Decrease in global DNA methylation	2016	70
	Multi-walled carbon nanotubes (MWCNTs)	10–50 nm	2 mg kg^−1^ mouse for 24 h	C57BL/6 mice	Hypomethylation of the TNFα promoter, hypermethylation of the THY-1 promoter	2019	72
Changes in histone modifications	PVP-coated silver (Ag)	25 nm	1–8 μg ml^−1^ for 72 h	MEL mouse erythroleukemia cells	Reduction in the levels of global H3 methylation	2015	75
	Biopolymer coated arsenic trioxide (As_2_O_3_)	75 nm	50–100 g ml^−1^ for 24 h	LNCaP and PC-3 human prostate cancer cell lines	Decrease in H3K9me, increase in H3S10 and H3K14ac	2016	77
	Zinc oxide (ZnO)	<100 nm	20–50 μg ml^−1^ for 24 h	HaCaT human epidermal keratinocytes	Increase in H3K9me2, decrease in H4K5ac	2016	78
	Silver (Ag)	<0.1 μm	1 mg ml^−1^ for 10 h	A549 human alveolar epithelial cells	Increase in H3S10 phosphorylation	2019	76
Changes in miRNAs expression	Cadmium telluride (CdTe)	<3 nm	15–45 mg ml^−1^ for 12–24 h	NIH-3T3 murine fibroblasts	Changes in the expression of 51 miRNAs	2011	82
	Silicon dioxide (SiO_2_)	70 nm	One injection of 10–20–40 mg kg^−1^	BALB/c mice	Increase in miR-122 and miR-192	2013	85
	Silver (Ag)	<100 nm	0.2 mg l^−1^ for 24 h	Human Jurkat T cell	Changes in the expression of 63 miRNAs	2014	79
	Titanium dioxide (TiO_2_)	38 nm	20–50–100 μg ml^−1^ for 24 h	A549 human alveolar epithelial cells	Downregulation of miRNA-21 and miRNA-30a	2017	81

## NPs for delivery of epi-drugs: the Yang of epigenetics in the field of NPs

Alterations of epigenetic mechanisms are involved in the etiology and in the progression of several diseases including, developmental disorders, cancer, neurological disorders and cardiovascular diseases (*e.g.*, heart failure and atherosclerosis).^8–10^ In cancer cells, for example, the loss of DNA methylation in heterochromatic regions causes both an increase of chromosomal instability, and a stochastic expression of gene content within these regions. These two events underlying tumor-cell heterogeneity are one of the main causes of chemoresistance of tumors.^34,87^ In neurological disorders, the involvement of epigenetic mechanisms was described in different aspects of the development of these diseases: mutations in epigenetic players can trigger several neurodevelopment disorders. The best known case is RETT syndrome, a severe neurodevelopmental disorder that affects mostly women, that is caused by mutations in the gene encoding methyl-CpG binding protein (MECP2), by mediating the effects of environmental risk factors for neurodevelopment disorders.^88,89^ Finally, recent findings showed that there are several underlying epigenetic mechanisms contributing to cardiovascular disease: in heart failure, the transcription program underlying cardiac hypertrophy is the result of alterations to the epigenome, and in atherosclerosis, epigenetic lesions contribute to atherosclerotic plaque development and progression.^90,91^

**Fig. 6:**
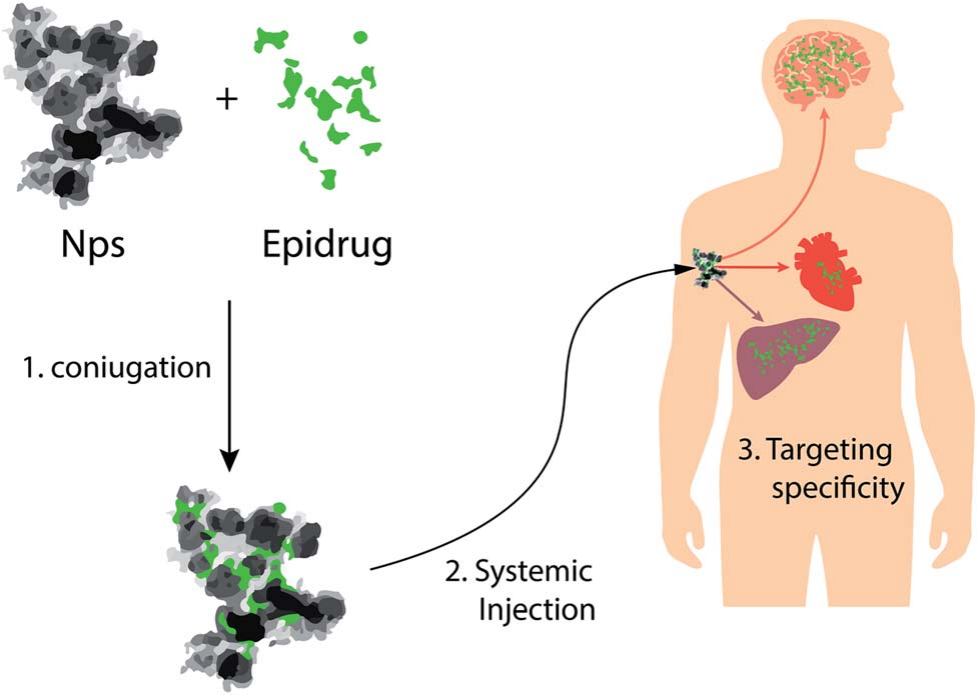
NPs as vectors for delivery of epi-drugs: schematic representation of NPs that can be used for tissue-specific delivery of epi-drugs.

Since epigenetic mechanisms are reversible, for many of these diseases, several therapeutic strategies have been proposed, based on the concept that epi-drugs can interfere with epigenetic changes responsible for disease, restoring the correct epigenetic landscape in diseased cells^12^ (Fig. 6). Although the use of these drugs was proposed for the treatment of several diseases, at the moment, the US FDA has approved only three types of epigenetic drugs: DNA methylation inhibitors (iDNMTs), histone deacetylase inhibitors (iHDACs) and histone methyltransferase inhibitors (iEZH2s), for the treatment of some tumors^92,93^ (Table 2).

**Table 2:** List of epigenetic drugs approved by the US FDA to date

Epi-drug name	Active ingredient	Formula	Epigenetic target	Clinical use	Year of approval
Vidaza	Azacitidine	C_8_H_12_N_4_O_5_	DNMT inhibitor	Myelodysplastic syndrome	2004
Dacogen	Decitabine	C_8_H_12_N_4_O_4_	DNMT inhibitor	Myelodysplastic syndrome	2006
Zolinza	Vorinostat	C_14_H_20_N_2_O_3_	HDAC inhibitor	Cutaneous T-cell lymphoma	2006
Istodax	Romidepsin	C_24_H_36_N_4_O_6_S_2_	HDAC inhibitor	Cutaneous T-cell lymphoma	2009
Beleodaq	Belinostat	C_15_H_14_N_2_O_4_S	HDAC inhibitor	Refractory peripheral T-cell lymphoma	2014
Farydak	Panobinostat lactate	C_21_H_23_N_3_O_2_·C_3_H_6_O_3_	HDAC inhibitor	Multiple myeloma	2015
Tazverik	Tazemetostat hydrobromide	C_34_H_44_N_4_O_4_·HBr	EZH2 inhibitor	Epithelioid sarcoma	2020

The major limitation of epigenetic therapy is the possibility of side-effects due to the fact that many targets of epi-drugs are ubiquitously expressed, and that some epi-drugs had a poor bioavailability, low stability and a short half-life. For instance, suberoylanilide hydroxamic acid (SAHA), known also as vorinostat (VOR), is a potent inhibitor of HDACs belonging to the class I of HDACS, whose use was approved for the treatment of cutaneous T-cell lymphoma by the FDA. However, its limitations regarding poor solubility and permeability reduces its clinical potential.^94^

These problems could be overcome with the generation of “smart epi-drugs” able to, on one hand, release the epi-drugs only in diseased cells, and on the other, to improve their pharmacokinetics. In the generation of “smart epi-drugs”, possible advances can emerge from the field of NPs. Indeed, in recent years, a fair number of biocompatible NPs have been developed^95,96^ with physical and chemical characteristics that make them adept to carrying drugs to the site of interest and control their release. These NPs bind the drug on their external surface, or internalize it, protecting them from premature activation. The use of NPs for this purpose has also made it possible to improve the pharmacokinetics (absorption, distribution, metabolism and elimination) and the specificity of the drugs, reducing the amount of drug required, and its side effects.^97–99^

To date, the most widely used nanoparticles as drug-delivery systems are polymer-based NPs, lipid-based NPs, and inorganic NPs. Although these nanoparticles guarantee a prolonged and controlled release of drugs, some of them, such as liposomes, have the disadvantage of being easily recognized by the reticuloendothelial system (RES) and, therefore, eliminated.^100^ Indeed, as soon as nanoparticles enter an organism, they are coated with a series of proteins, including opsonins, which modify their characteristics and facilitate their elimination. It is, therefore, of fundamental importance that the drug-delivery systems remain in circulation for longer time-periods without being opsonized and, therefore, phagocytosed. For this reason, a series of coatings have been developed in recent years, which are capable of avoiding the opsonization of nanoparticles, allowing them to evade the immuno-surveillance system and increase blood circulation half-life. It is possible to group these coatings into two categories: synthetic polymers (*e.g.*, PEG, POX and polyzwitterions) and “self-markers” (*e.g.*, CD47).^101^

Escaping the RES recognition and allowing a long circulation time is very important, for example, in order to transport the nanoparticles to tumors exploiting the abnormal tumor abnormal vasculature and lack of normal lymphatic system (enhanced permeability and retention – EPR-effect). In order to take advantage of the EPR effect in tumors, it is also necessary to create nano-drugs smaller than 200 nm. The size of the nano-drugs is important for permeabilization into the tumor, as nanoparticles that are too large would not be able to pass through the fenestrations of the tumor vessels which, generally, have dimensions between 200–800 nm. On the other hand, nano-drugs smaller than 6 nm would be easily directed to renal excretion, without having the time to act at the tumor level.^102,103^

The physico-chemical characteristics of the nanoparticles are therefore very important to effectively deliver the drugs to the site of interest, but also to allow a controlled release. For example, polylactic acid (PLA) NPs were proposed as a delivery system for drugs used for treatment of local dermatotherapies: PLA NPs destabilize in contact with sebum and, in this way, release their load exclusively at the level of the hair follicles and sebaceous glands.^104^

It was also proposed that the use of magnetic NPs could be used to deliver drugs in the specific tissue affected by the disease, by applying a magnetic field to it. The efficacy of this drug delivery system has been shown, for the first time, with dimercaptosuccinic acid (DMSA)-coated magnetic NPs loaded with IFN-γ, an anti-tumorigenic cytokine able to promote the activation and the infiltration of tumor-specific T-cells and macrophages in the tumor, and to inhibit tumor angiogenesis. In mouse models of cancer, these NPs can be targeted to the tumor tissue by application of an external magnetic field. This delivery strategy allowed a greater release of IFN-γ in the tumor site compared to what was found in mice treated with IFN-γ not conjugated with NPs: this leads to a more efficient immune response against the tumor and a major decrease in the tumor growth.^105^

A more sophisticated use of magnetic NPs is to combine them with materials able to release the drug in a controlled manner in response to stimuli that can be external (*e.g.*, magnetic field) or internal to organisms.^106^ An example of internal stimuli for release of drugs comes from superparamagnetic iron oxide NPs coated with folic acid (DOX@FA-SPIONs) and loaded with doxorubicin, an anticancer drug that blocks cell proliferation by inhibiting topoisomerase II, an enzyme required for DNA replication. This NP efficiently releases the drug only at acidic pHs: 90% of the doxorubicin is released at pH 5, while less than 20% is released at physiological pHs (7.4) in 48 h, conditions typical of tumors. This characteristic, together with the magnetic properties of NPs that permits them to accumulate it in the tumor through a magnetic field, have allowed researchers to obtain a nano-carrier that is very efficient in the delivery of anti-cancer drugs to tumors, with reduced toxicity, as shown by experiments carried out in MCF-7 breast cancer xenografts in nude mice.^107^ However, the possibility of finding internal stimuli that change so drastically in the diseased tissue for use as a trigger of drug release is quite rare, especially for those diseases in which drastic environmental and biochemical changes to the tissue have not occurred. For this reason, the use of external stimuli as activators of drug release seems more promising. Exploiting the property of magnetic NPs to produce heat when exposed to alternating magnetic fields, nanovectors, whose drug release depends on increase of temperature induced by alternating magnetic fields, were generated. Using this strategy, nanovectors were obtained with specific delivery and controlled drug release.^106^

The possibility of using NPs for generating “smart” epigenetic drugs is mainly supported by studies in the cancer field. Polymeric NPs functionalized with histone deacetylase inhibitors (iHDACs) led to an optimized release of iHDACs in mesothelioma cancer, resulting in an 80% reduction in the weight of the tumor without any type of toxicity.^108^ Liposomes have also been used as nano-carriers of epi-drugs: polyethylene glycol (PEG)-functionalized liposomes transport and release some anti-tumor drugs more efficiently, including the HDAC inhibitors SAHA, LAQ824, CG1521, PXD101 and TSA. Moreover, some of these inhibitors, such TSA, CG1521 and PXD101, when encapsulated with liposomes, increased their anti-tumoral activity against several solid tumors, including breast cancer.^109^ Moreover, PEG-liposome increases the solubility of the epigenetic drugs VOR and LAQ 824, and has a drug stability of one month at 4 °C.^110^ Also, dendrimers, that are NP polymer molecules made of a central core from which branches originate, have been used for the delivery HDACi in tumors without inducing toxic effects in non-target tissues. Using a cancer cell model, it was demonstrated that HDACis conjugated to dendrimers, unlike the non-conjugated drug, did not act against the tumor-associated macrophages, reducing the drug resistance mediated by these cells.^111,112^

Moreover, inorganic NPs were used as nanocarriers for epigenetic drugs. For example, gold NPs conjugated with polyethylene glycol are able to transport and release some HDACis (*e.g.*, vorinostat) in the tumor, leading to a decrease in tumor growth. These nanocarriers have the advantage of easily crossing the endothelium, thus allowing the drug to spread rapidly through the circulation. Moreover, the silica NPs MCM-41-VOR and MSMs increase the solubility and permeability of vorinostat, in particular when amino and phosphonate groups were added to them. Assays carried out in colorectal (HCT 116) and cutaneous T cell lymphoma (CTCL) cell lines showed that the drug incapsulated in amino-functionalized silica NPs had better anti-tumour activity than the free drug.^94^

The use of NPs has also been proposed for the delivery of drugs able to modulate the level of miRNAs, such as miRNA mimics or miR inhibitors/anti-miRNAs, in cancer. miRNA mimics, having an identical sequence to the endogenous mature miRNA, are used to increase the level of miRNA when it is down-regulated in diseases, while miR inhibitors/anti-miRNAs have a complementary sequence to the mature miRNA, and are used to decrease the level of miRNAs that are up-regulated in disease.^113^ The limitations of these drugs lie in their instability in the blood due to their rapid degradation or inactivation by blood nucleases, and non-specific cellular uptake, which can make these drugs highly toxic. To overcome these problems, the use of NPs as carriers of these drugs has been proposed. For example, intranasal administration of gold-iron oxide NPs loaded with both miR-100 (whose under-expression contributes to tumorigenesis) and anti-miR-21 (a potent overexpressed oncomiR in glioblastoma) in nude mice enhanced the effects of chemotherapy at the level of glioblastoma cells,^114^ whereas intravenous administration of gold NPs functionalized with miR-182 (which acts as a tumor suppressor by controlling the expression of oncogenes deregulated in glioblastoma) caused a reduction in tumor burden and increased animal survival in glioblastoma xenografts.^115^ Also lipid NPs were used to deliver several miRNAs such as miR-29b, miR-634, miR-660, miR-34a and let-7b, whose down-regulation promotes cancer progression of different human tumors. The effectiveness of these nanocarriers was demonstrated using mice models of cancer. For example, cationic lipid NPs loaded with miR-29b increased the expression of this miRNA by 5-fold in lung tumor cells, and neutral lipid emulsion complexed with miR-34a or le7-b inhibits lung tumor growth in mice.^116^ Interestingly, polyamidoamine (PAMAM, dendrimers of repetitively branched amide and amine unit) was used to deliver the miR-let-7a and miRNA-122 to neuroblastoma tumors and liver cancer, respectively, in mice models of these cancers. In order to have a tumor-specific miR delivery, recently, a PAMAM was obtained that was able to release its cargo of miR-122, a miRNA whose down-regulation is involved in the liver tumorigenesis by exploiting the acidic nature of the tumor microenvironment. This nano-carrier was used to deliver the miR-122 in liver cancer, in mice models of this tumor.^117^ The use of nanocarriers to deliver miRs is not limited only to the tumor field, but their use was also proposed for the treatment of other diseases such as cardiovascular diseases, immune diseases and neurological diseases.^118,119^ Among these, the most promising applications of these therapeutic strategies is for some cardiovascular diseases such heart failure, myocardial infarction and atherosclerosis, where the deregulation of various miRs is a key event of these diseases. Heart failure is characterized by myocardial remodeling that compromises heart function. Underlying this remodeling, cardiac hypertrophy and cardiac fibrosis occur, which are pathological processes that result from gene expression and epigenetic changes occurring in cardiomyocytes and cardiac fibroblasts, respectively.^10^ Clearly defining such changes could play an important role in utilizing several miRs, including miR-133, whose down-regulation in cardiomyocytes is a key step in defining the cardiac hypertrophy phenotype, as therapeutic targets.^120^ Restoring the correct expression of this miR in cardiomyocytes of mice subject to transverse aortic constriction, a surgical model of pressure overload-induced cardiac hypertrophy and heart failure, could be important in improving cardiac function. In order to develop a miR-based therapy for this disease, it was proposed that propagating miR-133 to the heart with a certain selectivity could be achieved using calcium phosphate (CaP) NPs. This selectivity was obtained by synthesizing these NPs with citrate, that in addition to stabilizing the CaP-NPs at the early stages of crystallization, gives them a negative surface charge. This chemical property makes them more susceptible for uptake by polarized tissues such as the heart. Other important features of these NPs are biocompatibility, bioresorbability and biodegradability, since their structural and chemical properties are similar to those of the mineral components of bones. Moreover, the acidic pH of endosomes dissolves these NPs into their ionic constituents, allowing the release of miRs into the cytoplasm without any residual accumulation.^121^

Myocardial infarction (MI) is accompanied by an inflammatory reaction that contributes to defining cardiac remodeling leading to HF. This immune response plays a key role in the infiltration of neutrophils and macrophages in the infarcted area of the myocardium. Interference with this inflammatory process could improve heart function during an MI. In order to achieve this, NPs were generated that are able to deliver miRs to macrophages, that are in turn, able to block this pathological process. For example, acid-degradable polyketal NPs loaded with three miRNAs (miR-106b, miR-148b and miR-204) were able to down-regulate the expression of NOX2 in macrophages: up-regulation of these miRNAs in these cells during myocardial infarction contributes to an increase of reactive oxygen species (ROS), promoting cardiac remodeling leading to HF.^122^ Interestingly, hyaluronan-sulfate (HAS) NPs loaded with miR-21 were used to increase the level of this miR in macrophages, thus improving heart function.^123^ The use of NPs loaded with miRs for the treatment of atherosclerosis, an inflammatory disease of the arterial system that can lead to myocardial infarction, ischemic stroke and peripheral arterial disease, was also proposed. During the formation of atherosclerotic plaques, endothelial cells are highly involved in pathogenesis: once endothelial cells are chronically activated by a combination of turbulent blood flow, lipid accumulation in the vessel wall and exposure to pro-inflammatory cytokines (*e.g.*, TNF-α, IFN-γ and IL-1β), promotes the recruitment and maintenance of inflammatory cells. Underlying the activation of endothelial cells is a reprogramming of gene expression in which miRs play a key role in their definition. Thus, miRNA-based therapies coupled with NPs were proposed as a promising therapeutic target for this disease. Regarding this, lipid NPs were generated that were able to deliver anti-miR-712, whose up-regulation in endothelial cells promotes their activation, specifically at the inflamed regions of atherosclerotic lesions. The specific uptake was obtained by generating lipid-based NPs made by two layers: the outer layer was composed of neutral lipids, containing a peptide (with the sequence VHPKQHR), which provides specificity for internalization in inflamed endothelial cells, while the inner layer was composed of cationic lipids capable of encapsulating an aqueous solution containing the anti-miRNA within the NPs.^94^

NPs have also been proposed to improve the selectivity of current epigenetic drugs. Indeed, one of the major limitations of epigenetic drug therapy is its toxicity, which is mainly due to the ubiquitous expression of epigenetic enzymes. In order to overcome this problem, the design of epigenetic drugs that are able to simultaneously inhibit two or more epigenetic enzymes that are consequently deregulated in diseased cells could prove to be a very promising target in creating selective epigenetic drugs for the pathways involved in disease onset. Taking advantage of the chemical compounds regulating epigenetic enzymes, the use of NPs could be exploited to combine different epi-drugs to improve their therapeutic effect and selectivity. The proof-of-concept of this strategy comes from a study where the action of the anti-cancer drug decitabine (DAC), an inhibitor of DNA hypermethylation, was improved when combined with doxorubicin (DOX), through the conjugation of these two drugs with biodegradable MPEG-*b*-PLA NPs, in order to obtain NPDACs and NPDOXs, respectively. The administration of these NPs in an MB-MDA-231 xenograft murine model showed that they were able to deliver these drugs more efficiently into the engrafted breast, thus promoting the inhibition of growth of breast cancer cells through the induction of apoptosis of tumor cells.^124^ The possibility of combining more than one drug into NPs has allowed for the possibility to build nanocarriers capable of carrying a combination of epigenetic drugs coupled with a second type of drug (*e.g.*, inhibitor of cell cycle progression) in order to amplify the therapeutic effects. For example, the drug paclitaxel (PTX), which is one of the first-line chemotherapeutic drugs used for treatment of ovarian cancer, currently possesses certain limitations related to drug resistance as a result of activation of the EGFR/ERK pathway. In order to overcome this problem, NPs that simultaneously deliver PTX and miR-7, suppressors of the EGFR/ERK pathway, were developed.^125^ NPs co-transporting doxorubicin (DOX) and miR34a were also generated in order to obtain a more pronounced anti-tumor effect of DOX: inasmuch, miR34a suppresses the expression of genes involved in drug resistance and BCL2, a pro-oncogene that has anti-apoptotic activity.^126^

To date, several studies have therefore demonstrated the efficacy of nanoparticles as miRNA delivery systems, however there are still few studies that have explored the possibility of using NPs as delivery systems for HDAC and DNMT inhibitory drugs (Table 3). In the future, further investigation is necessary regarding the use of biocompatible NPs as delivery systems for epi-drugs in order to make the best use of these drugs in the context of pharmacotherapy. In fact, due to the possibility of carrying nanoparticles to the locus of interest through active or passive targeting, it is possible to prevent epigenetic drugs from acting systemically, thus limiting their toxicity exclusively at the level of the locus of interest and increasing their safety.

**Table 3:** List of the main studies regarding the use NPs for epi-drugs delivery

Drug delivery system	Biological model	Clinical use	Year	Reference
PEG-liposomes loaded with HDACi	MCF-7, T47-D A 1–2, SKBr-3 and MDA-MB-231 cell lines	Breast cancer therapy	2010	109
Cationic lipoplexes loaded with miR-29b	Athymic nude mice	Lung cancer treatment	2013	116
DOX-miR-34a co-loaded HA-CS NPs	BALB/c nude mice	Breast cancer therapy	2014	126
Gold NPs functionalized with miR-182	SCID mice injected with U87MG or GIC-20 cells	Glioblastoma treatment	2015	115
MPEG-*b*-PLA NPs loaded with DAC or DOX	MB-MDA-231 xenograft murine model	Breast cancer therapy	2015	124
Polymeric NPs loaded with HDACi	C57BL/6 mice	Tumor therapy	2016	108
Silica NPs MCM-41-VOR (HDACi)	HCT116 and cutaneous T-cell lymphoma cell lines	Colon cancer and cutaneous T-cell lymphoma treatment	2018	94
Hyaluronan-sulfate NPs loaded with miR-21	C57BL/6 mice	Post-myocardial infarction and heart failure therapy	2018	123
PTX/miR-7 NPs	BALB/c nude mice	Ovarian cancer therapy	2018	125
GIONs loaded with miR-100 and anti-miR-21	Nude mice	Glioblastoma treatment	2019	114

## Conclusions

We believe that intersecting epigenetics and nanotechnology will push both fields forward. On one side, the assays normally used to define nanotoxicity are not able to evaluate latent toxicity. The complexity of the epigenetic language and the heritability of epigenetic marks makes epigenetics a relevant mechanism for this type of toxicity. Clarifying the impact of NPs on the epigenome will allow to define latent toxicity and thus describe more accurately the toxicity of these nanomaterials. Indeed, NPs could cause epigenetic lesions that, although may not affect gene expression in the short term, could accumulate over time with other epigenetic lesions, leading to an alteration of gene expression and, consequently, of phenotype. The full understanding of the effects of NPs on the epigenome will require epigenetic studies not only able to correlate the epigenome with the transcriptional status of cells, but also capable of investigating the mechanisms by which NPs cause these epigenetic lesions.

On the other side, NPs are emerging as potential vectors for epigenetic drugs. However, the nanocarriers currently available for epi-drugs are only available for certain types of cancers, and have low selectivity. Therefore, there is an urgent need for nanocarriers capable of delivering epigenetic drugs with greater selectivity, and more importantly, to cells involved in other diseases, where the therapeutic potential of epigenetic therapies has been shown (*e.g.*, cardiovascular and neurological disorders). Regarding this, a great opportunity will come from the functionalization of the surface of NPs with molecules that allow a selective absorption by the target cells. Indeed, although these delivery strategies have shown quite a modest targeting performance, we think that a better characterization of pathways involved in cell specific uptake, as well as the use of magnetic NP systems, could help to overcome this limitation.^22^

## Conflicts of interest

No conflicts of interest, financial or otherwise, are declared by the authors.

## Supplementary Material
